# Detection of Low-Level Fosfomycin-Resistant Variants by Decreasing Glucose-6-Phosphate Concentration in Fosfomycin Susceptibility Determination

**DOI:** 10.3390/antibiotics9110802

**Published:** 2020-11-12

**Authors:** Guillermo Martín-Gutiérrez, Fernando Docobo-Pérez, Jose Manuel Rodríguez-Martínez, Alvaro Pascual, Jesús Blázquez, Jeronimo Rodriguez-Beltrán

**Affiliations:** 1 Unidad de Enfermedades Infecciosas, Microbiología y Medicina Preventiva, Hospital Universitario Virgen del Rocio, 41013 Seville, Spain; 2 Instituto de Biomedicina de Sevilla (IBiS), Hospital Universitario Virgen del Rocío/CSIC/Universidad de Sevilla, 41013 Seville, Spain; fdocobo1@us.es (F.D.-P.); jmrodriguez@us.es (J.M.R.-M.); apascual@us.es (A.P.); 3 Red Española de Investigación en Patología Infecciosa (REIPI), Instituto de Salud Carlos III, 28029 Madrid, Spain; 4 Departamento de Microbiología, Universidad de Sevilla, 41013 Seville, Spain; 5 Unidad de Enfermedades Infecciosas, Microbiología y Medicina Preventiva, Hospital Universitario Virgen Macarena, 41009 Seville, Spain; 6 Centro Nacional de Biotecnología, Consejo Superior de Investigaciones Científicas, 28006 Madrid, Spain; 7 Department of Microbiology, Hospital Universitario Ramon y Cajal, Instituto Ramón y Cajal de Investigación Sanitaria (IRYCIS), 28034 Madrid, Spain; jeronimo.rodriguez@salud.madrid.org; 8 Spanish Consortium for Research on Epidemiology and Public Health (CIBERESP), Instituto de Salud Carlos III, 28029 Madrid, Spain

**Keywords:** glucose-6-phosphate, fosfomycin resistance, *Escherichia coli*, MIC, urinary tract infection

## Abstract

Mutations that confer low-level fosfomycin resistance (LLFR) but not clinical resistance in *Escherichia coli* are increasingly reported. LLFR strains can become clinically resistant under urinary tract physiological conditions or may act as gateways for highly resistant subpopulations by the selection of additional LLFR mutations. Nevertheless, most LLFR strains are impossible to detect under routine fosfomycin susceptibility determinations. Here, we have explored the possibility of detecting LLFR variants by reducing glucose-6-phosphate (G6P) concentration in fosfomycin susceptibility testing for *E. coli* strains. As a proof of concept, fosfomycin minimal inhibitory concentrations (MICs) and disk diffusion susceptibility tests were performed for *E. coli* strain BW25113 and 10 isogenic derivatives carrying the most prevalent LLFR chromosomal mutations (∆*uhpT*, ∆*glpT*, ∆*cyaA*, and ∆*ptsI*) and their double combinations. Whereas standard G6P concentrations detected only ∆*uhpT* single and double variants, assays with reduced G6P detected all LLFR variants. In addition, G6P levels were determined to be ≤5 µg/mL in urine samples from 30 patients with urinary tract infection (UTI) caused by *E. coli* and 10 healthy volunteers, suggesting that most bacterial cells in uncomplicated UTIs are facing fosfomycin under low G6P concentration. Reducing G6P allows for the detection of LLFR variants, which may suppose a risk for future resistance development, especially in UTIs.

## 1. Introduction

Fosfomycin is a phosphonic acid derivative produced by a broad variety of *Streptomyces* and *Pseudomonas* species [1]. This natural antibiotic has been used widely as a first-line agent for the empirical treatment of urinary tract infections (UTIs), with fosfomycin trometamol as the preferred formulation for oral administration [2]. In *Escherichia coli*, fosfomycin is transported actively into the bacterial cytoplasm via GlpT and UhpT transporters. UhpT is a chemiosmotic transporter that catalyzes intracellular accumulation of glucose-6-phosphate (G6P) by exchange with internal inorganic phosphate [3]. Expression of *uhpT* is precisely regulated by the response regulators UhpA and UhpB that, after recognition of extracellular G6P by the constitutively expressed sensor UhpC, activate expression of UhpT [1,4]. G6P increases expression of the *uhpT* gene, increasing fosfomycin uptake and thus fosfomycin susceptibility [5]. Mutations in *cyaA* or *ptsI* genes produce a decrease in intracellular levels of Cyclic adenosine monophosphate (cAMP) [2], which is necessary for full expression of the fosfomycin transporters GlpT and UhpT, leading to a reduced fosfomycin uptake [1,4]. The presence of chromosomal loss-of-function mutations in these genes and some of their combinations confer low-level fosfomycin resistance (LLFR), but not clinical resistance according to international guidelines [6]. However, although the presence of LLFR mutations yields a fosfomycin-susceptible phenotype, they may act as gateways for highly resistant subpopulations by the selection of additional LLFR mutations [7,8]. Furthermore, the presence of LLFR mutations might confer clinical resistance under specific environments, such as urinary tract physiological conditions [9]. Therefore, detecting LLFR variants is crucial to establish their prevalence and estimate the risk of resistance development during fosfomycin treatment.

From a physiological point of view, G6P is thought to be coupled with glycolysis in most human tissues [5]. The exact G6P concentration in vivo varies across tissues and is difficult to determine experimentally. It has been shown that the addition of 25µg/mL of G6P to culture medium produces fosfomycin minimal inhibitory concentration (MIC) results that correlate well with susceptibility data obtained with tissue homogenates for several bacterial species such as *E. coli*, *Klebsiella* spp. and *Proteus mirabilis* [10], suggesting that bacteria may encounter some G6P when infecting tissues. Moreover, fosfomycin effectiveness in reducing intracellular *E. coli* was determined to be low [11], suggesting a limited intracellular availability of fosfomycin. Thus, during uncomplicated UTIs, probably most E. coli cells facing fosfomycin are growing planktonically in the urine of patients or attached to the urothelium. Interestingly, urine seems to be free of glucose, as the kidneys normally reabsorb all glucose into the bloodstream [12]. It is thus unclear whether this urinary pathogen encounters much G6P during uncomplicated urinary tract infections, where tissue damage is minimal. It is possible, therefore, that the majority of *E. coli* cells in uncomplicated UTIs are treated with fosfomycin under low G6P concentrations.

As indicated above, fosfomycin susceptibility is routinely tested in the presence of G6P, whose concentration was standardized to 25 µg/mL to increase reproducibility of fosfomycin susceptibility testing [13]. Following this criterion, both the Clinical and Laboratory Standards Institute (CLSI) and the European Committee on Antimicrobial Susceptibility Testing (EUCAST) recommend MIC of fosfomycin to be determined by the agar dilution method in the presence of 25 µg/mL G6P and susceptibility by disk diffusion tests using 200 µg fosfomycin disks containing 50 µg G6P [14,15]. Under these premises, we hypothesized that the presence of high concentration of G6P in routine MIC determinations may hinder the detection of *E. coli* strains carrying mutations that lead to LLFR, other than those affected by G6P, such as *uhpT*. To test this hypothesis, we analyzed the concentration of G6P in urine samples from UTI patients and healthy volunteers. We subsequently studied a set of isogenic strains bearing the most prevalent chromosomal mutations that decrease fosfomycin susceptibility and several combined mutations [6] under different G6P concentrations.

## 2. Results

To study G6P levels during UTI, we collected urine samples from 30 patients with UTI symptoms and from 10 healthy volunteers, which we analyzed in a specific enzymatic assay. All samples tested had G6P values lower than the detection limit of the procedure (≤5 µg/mL), independently of their origin (UTI or control) (data not shown). Then, we tested fosfomycin susceptibility of isogenic strains harboring different LLFR mutations with different G6P concentrations.

Table 1 shows fosfomycin MIC values for the isogenic strains obtained on Mueller–Hinton (MH) agar with G6P at 25 µg/mL (recommended concentration), 5 µg/mL (detection limit for our G6P detection assay), and without G6P (a possibility, on the basis of our results). Moreover, disk diffusion results obtained by using recommended fosfomycin disks (fosfomycin 200 µg with G6P 50 µg) and those obtained by reducing G6P concentration (fosfomycin 200 µg with 25 µg, 15 µg, and without G6P) are shown. At 25 µg, the G6P concentration used routinely in clinical laboratories, the only mutation that conferred clinical resistance to fosfomycin by agar dilution was Δ*uhpT* and its double mutant derivatives *(*Δ*uhpT*-Δ*ptsI,* Δ*uhpT-*Δ*cyaA,* Δ*uhpT-*Δ*glpT).* All other mutations did not reach the breakpoint for clinical resistance to fosfomycin. The MIC value for the fosfomycin transporter mutant ∆*glpT* was similar to that of the wild type strain in this condition.

These results suggested that the effect of mutations in genes that confer LLFR (other than *uhpT*) is underestimated when the recommended G6P concentration is used. To test this possibility, we performed additional MIC and disk diffusion determinations without G6P or at lower concentrations (Table 1). As anticipated, ∆*uhpT* susceptibility remained constant, independently of the presence or absence of G6P. In MH agar supplemented with 5 µg/mL G6P, fosfomycin MIC values for Δ*uhpT* and Δ*glpT* remained at 64 µg/mL, while those for Δ*cyaA* and Δ*ptsI* strains were higher (512 and 256 µg/mL, respectively). Increased MIC values were observed for all double mutants. In the absence of G6P, further MIC increases were found for the double mutants Δ*cyaA*-Δ*glpT* and Δ*ptsI-*Δ*uhpT*. When G6P was not present, fosfomycin resistance could thus be achieved by inactivating mutations in genes other than *uhpT*. Similar results were obtained by the disk diffusion methodology, where only the disks with 15 µg of G6P uncovered the presence of all LLFR mutations related with fosfomycin resistance.

## 3. Discussion

Although urine of healthy humans is normally glucose-free, the G6P level in urine of UTI patients is not well known. During UTI, *E. coli* is capable of forming intracellular bacterial communities within the superficial cells of the bladder, where G6P concentration is supposed to be high [10,16]. The activity of bacterial virulence factors damages the bladder epithelium, releasing *E. coli* cells into the bladder lumen [16] and leading to the liberation of intracellular molecules such as G6P. However, our results indicate that G6P concentration in human urine is likely very low, even in patients with UTIs, differing markedly from the G6P concentration recommended for use in fosfomycin MIC determinations.

The results obtained by MIC and disk diffusion indicate that addition of a high G6P concentration in routine MIC determinations might not identify strains that encode LLFR-conferring mutations in genes other than *uhpT*. A recent study sequenced inner colonies that appeared in a collection of 649 multidrug-resistant *E. coli* after fosfomycin disk susceptibility testing in the presence of G6P. All fosfomycin-resistant inner mutants were defective in *uhpT* or its regulators [17]. This supports the idea that routine fosfomycin MIC determinations using the recommended G6P concentration are particularly biased towards detecting Uhp system mutants. This situation can be further compounded as most clinical microbiology laboratories determine fosfomycin susceptibility by methods that are not recommended by EUCAST or CLSI criteria, such as automated methods or gradient strips (E-test).

Besides fosfomycin resistance, it was recently shown that *cyaA* deletion in uropathogenic *E. coli* causes a metabolic perturbation that leads to a notable increase in the production of persister cells, which are tolerant to β-lactam antibiotics [18]. Therefore, the presence of some LLFR mutations (undetected by standard procedures) could be linked to relapsing and recurrent UTIs. This situation, together with the low concentration of G6P in urine might provide an ideal scenario for the selection of these LLFR strains during UTI treatment with fosfomycin. Moreover, some LLFR strains not considered clinically resistant according to international guidelines [6,19] can directly confer clinical resistance under urinary tract physiological conditions [9] or act as gateways for highly resistant bacterial populations with additional mutations.

Fosfomycin susceptibility is relatively high but varies on the basis of bacterial species and by geographical regions [20]. In Spain, for instance, a rise in fosfomycin resistance rate has been reported, which was attributed to the increased use of fosfomycin in community-acquired UTI [21]. Similar results have been observed in other countries, such as Israel, where Peretz and colleagues [22] observed a susceptibility rate of 77% in 1503 Gram-negative urinary isolates. A review performed by Falagas and colleagues showed declining fosfomycin activity in (extended-spectrum beta-lactamases) ESBL-producing *E. coli* and *Klebsiella pneumoniae* (96.8% and 81.3%, respectively) [20]. However, other countries still show very low rates of fosfomycin resistance, even in multi-drug-resistant strains [23]. This fact, along with the presence of synergistic interactions with many antibiotics, as well as the total absence of cross resistance with other antibiotics, makes fosfomycin a good partner drug in clinical practice against multi-drug-resistant pathogens [24].

Despite the limited prevalence of fosfomycin-resistant isolates from UTI patients, the increased use of this antibiotic could amplify the proportion of LLFR and resistant variants, as has been the case for most antimicrobial drugs. The effect G6P concentration and LLFR mechanisms, typically not analyzed in large cohorts of clinical strains, should be studied to determine the weight of each molecular mechanism on susceptibility according to the G6P concentration and the possible impact of LLFR on UTI eradication.

## 4. Material and Methods

### 4.1. G6P Determination in Urine from Patients with UTI

We determined the presence of G6P in 30 urine samples from patients infected with *E. coli* and 10 control samples from healthy volunteers. Midstream catch urine was collected in sterile tubes, transported under refrigeration, and processed within 4–6 h after sampling. Bacterial culture and identification were carried out according to standard hospital procedure. Urine samples included for G6P determination were selected by using systematic random sampling; bloody urine was excluded. G6P determinations were performed by enzyme assay using a G6P kit (MAK014; Sigma-Aldrich, Spain) following the manufacturer’s instructions. The study was approved by the Research Ethics Committee of the University Hospitals Virgen del Rocío and Virgen Macarena (Seville, Spain) (GMG-FOS-2017-01).

### 4.2. Strains and Media

Fosfomycin MIC with and without G6P were determined for *E. coli* strain BW25113 and for 10 isogenic derivatives with the most prevalent fosfomycin chromosomal mutations (∆*glpT*, ∆*uhpT*, ∆*cyaA*, and ∆*ptsI*), single or combined, as constructed by Ballesteros-Téllez et al. [6], on the basis of the single mutants from the KEIO collection [25] (Table 1). Agar dilution method was performed on MH agar plates containing fosfomycin at different concentrations (from 0.25 to 2048 μg/mL) and supplemented with G6P at 25 μg/mL, 5 μg/mL, or no G6P.

### 4.3. MIC Determination

Fosfomycin susceptibility was determined in Mueller–Hinton (MH) by agar dilution (gold standard) and disk diffusion methods according to CLSI/EUCAST guidelines [14,15]. For the agar dilution method, bacterial cultures were adjusted to the 0.5 MacFarland standard and diluted 1:10 in saline solution. Then, 2 μL (approximately 10^4^ CFU) were spotted onto the agar surface. Once the spots on the agar were absorbed, the plates were incubated at 35 ± 2 °C for 16 h. The MIC was defined as the lowest antibiotic concentration that inhibited visible growth. Strains with a MIC value for the clinical strains of ≤4 μg/mL were considered fosfomycin-susceptible, between >4 and ≤32 μg/mL as LLFR (still susceptible), and >32 μg/mL as fosfomycin-resistant. For the disk diffusion method, disks containing 200 μg of fosfomycin with 50 μg, 25 μg, 15 μg, and without G6P were used. Blank disks (Oxoid) were impregnated with 25 μL of each fosfomycin–G6P concentration. Diameter (in millimeters) of the zone of complete inhibition was determined after 16–20 h of incubation at 35 ± 2 °C. MICs and disk diffusion tests were repeated 3 times for each strain. Final data are the modal value from 3 repetitions. *E. coli* ATCC 25922 (laboratory collection) was used as the MIC control strain.

## Figures and Tables

**Table 1:** Fosfomycin susceptibility results of *Escherichia coli* strains performed by agar dilution and disk diffusion methods.

Strain	Agar Dilution			Disk Diffusion (mm)			
	MH + G6P 25 μg/mL *	MH + G6P 5 μg/mL	MH No G6P	200 μg + G6P 50 μg *	200 μg + G6P 25 μg	200 μg + G6P 15 μg	200 μg
ATCC25922	2	16	32	30	27	24	**22**
BW25113	2	8	2	31	27	25	**22**
Δ*cyaA*	16	**512**	**512**	27	24	**22**	**0**
Δ*glpT*	2	**64**	**128**	29	**22**	**21**	**18**
Δ*uhpT*	**64**	**64**	**64**	**19**	**20**	**20**	**20**
Δ*ptsI*	8	**256**	**256**	28	**23**	**23**	**15**
Δ*glpT-*Δ*uhpT*	**256**	**128**	**128**	**14**	**15**	**15**	**15**
Δ*glpT-*Δ*ptsI*	8	**512**	**512**	**23**	**23**	**21**	**0**
Δ*cyaA-*Δ*glpT*	32	**256**	**1024**	28	24	**21**	**0**
Δ*ptsI-*Δ*cyaA*	32	**512**	**512**	26	24	**22**	**0**
Δ*uhpT-*Δ*cyaA*	**512**	**512**	**512**	**0**	**0**	**0**	**0**
Δ*ptsI-*Δ*uhpT*	**64**	**64**	**128**	**11**	**10**	**10**	**11**

Fosfomycin breakpoints for *E. coli* according to reference 14: minimal inhibitory concentration (MIC) > 32 = R; diameter mm: <24 = R. Resistant values are shown in bold. * Recommended methodology according to Clinical and Laboratory Standards Institute (CLSI) and European Committee on Antimicrobial Susceptibility Testing (EUCAST).
